# Orsay Virus Infection of Caenorhabditis elegans Is Modulated by Zinc and Dependent on Lipids

**DOI:** 10.1128/jvi.01211-22

**Published:** 2022-11-07

**Authors:** Luis Alberto Casorla-Perez, Ranya Guennoun, Ciro Cubillas, Bo Peng, Kerry Kornfeld, David Wang

**Affiliations:** a Department of Molecular Microbiology, Washington University in St. Louis, St. Louis, Missouri, USA; b Department Pathology & Immunology, Washington University in St. Louis, St. Louis, Missouri, USA; c Developmental Biology, School of Medicine, Washington University in St. Louis, St. Louis, Missouri, USA; University of Kentucky College of Medicine

**Keywords:** *Caenorhabditis elegans*, lipid regulation, Orsay virus, zinc, replication, *sbp-1*/*srebp-1*

## Abstract

Viruses utilize host lipids to promote the viral life cycle, but much remains unknown as to how this is regulated. Zinc is a critical element for life, and few studies have linked zinc to lipid homeostasis. We demonstrated that Caenorhabditis elegans infection by Orsay virus is dependent upon lipids and that mutation of the master regulator of lipid biosynthesis, *sbp-1*, reduced Orsay virus RNA levels by ~236-fold. Virus infection could be rescued by dietary supplementation with lipids downstream of *fat-6/fat-7*. Mutation of a zinc transporter encoded by *sur-7*, which suppresses the lipid defect of *sbp-1*, also rescued Orsay virus infection. Furthermore, reducing zinc levels by chemical chelation in the *sbp-1* mutant also increased lipids and rescued Orsay virus RNA levels. Finally, increasing zinc levels by dietary supplementation led to an ~1,620-fold reduction in viral RNA. These findings provide insights into the critical interactions between zinc and host lipids necessary for virus infection.

**IMPORTANCE** Orsay virus is the only known natural virus pathogen of Caenorhabditis elegans, which shares many evolutionarily conserved pathways with humans. We leveraged the powerful genetic tractability of C. elegans to characterize a novel interaction between zinc, lipids, and virus infection. Inhibition of the Orsay virus replication in the *sbp-1* mutant animals, explained by the lipid depletion, can be rescued by a genetic and pharmacological approach that reduces the zinc accumulation and rescues the lipid levels in this mutant animal. Interestingly, the human ortholog of *sbp-1*, *srebp-1*, has been reported to play a role for virus infection, and zinc has been shown to inhibit the virus replication of multiple viruses. However, the mechanism through which zinc is acting is not well understood. These results suggest that the lipid regulation mediated by zinc may play a relevant role during mammalian virus infection.

## INTRODUCTION

Viruses continue to be a global threat to human, animal, and plant health. Although significant advances have been achieved in understanding host-virus interactions, there are still many questions unanswered. Caenorhabditis elegans is an animal model with many genes and biological processes that are evolutionarily conserved in higher eukaryotes. Thus, many fundamental discoveries in this model organism have been extrapolated into humans, such as apoptosis, RNA interference (RNAi), and microRNAs (1–3). Likewise, in recent years, C. elegans has become a model to study virus-host interactions following the discovery of Orsay virus, the first known natural virus of this organism (4, 5). This *in vivo* infection system has enabled the identification of novel host factors required for viral infection (6–10). Further characterization of host-virus interactions by employing this genetically tractable model provides the opportunity to elucidate underlying mechanisms utilized by viruses to proliferate.

Orsay virus is a nonenveloped, single-stranded, positive-sense RNA virus related to viruses in the family *Nodaviridae* (4). The genome of Orsay virus is composed of two RNA segments, the first of which encodes an RNA-dependent RNA polymerase (RdRp) in the RNA1 segment (~3.4 kb). The RNA2 segment (~2.5 kb) encodes the viral capsid and a capsid-delta fusion protein that is generated by a ribosomal frameshifting mechanism (11). The Orsay capsid has a T=3 icosahedral symmetry with 60 trimeric surface spikes (12). In addition, a plasmid-based genetic reverse system was developed by generating transgenic animals harboring the Orsay virus cDNAs (13). Orsay virus infects primarily intestinal cells, which leads to morphological changes of the intestine including fusion of intestinal cells, induction of vesicles, and disappearance of nuclei (4, 14). Little is known about the host factors required for Orsay virus infection in C. elegans. A few genes, *sid-3*, *viro-2*, *nck-1*, *drl-1*, and *hipr-1*, essential for Orsay virus infection that act on early, prereplication stages of the virus life cycle have been identified (6, 9, 10).

Viruses depend on host cells to produce viral proteins, replicate their genome, and assemble infectious particles to complete their viral life cycle. The building blocks and energy required for viruses are provided by the host cell. RNA viruses exploit the membranes and intracellular lipids of the host during infection (15–18). One example is rotavirus, for which it was shown that colocalization of the lipid droplets (LDs) with the replication center and drugs that interfere with LD formation inhibited the viral RNA replication and production of viral progeny (19). In addition, it has been shown that LDs are important in replication for multiple viruses like hepatitis C virus (HCV) (20), dengue virus (21), picornaviruses (22, 23), noroviruses (24), SARS-CoV-2 (25–27), and DNA viruses like Marek’s disease virus (28). Understanding the mechanisms employed by viruses to disrupt and exploit lipid metabolism may provide means to develop countermeasures against viruses.

The sterol-regulatory-element-binding protein (*srebp*) is a transcription factor that belongs to the basic helix-loop-helix leucine zipper family and is important for the homeostasis of lipids in the cell. In mammals, there are two *srebp* genes, *srebp1* and *srebp2*. *srebp1* is mainly involved in the expression of fatty acid biosynthesis genes whereas *srebp2* is involved in cholesterol biosynthesis (29). During the maturation of these proteins, the newly synthesized *srebp* is located in the endoplasmic reticulum (ER) membrane as a precursor. When specific cellular lipids are low, the protein is transported to the Golgi complex and released by proteases. Then, the mature *srebp* is translocated into the nucleus, where it induces the transcription of more than 30 lipogenic genes (30). As *srebp* has a vital role in cellular lipid metabolism, many viruses have subverted this transcription factor according to their needs. For example, it has been described that HCV (31–34), coxsackievirus B3 (CVB3) (35, 36), and human cytomegalovirus (37, 38) promote the accumulation of lipids through induction of the *srebp* pathway. Likewise, it has been reported that multiple viruses require lipids for efficient infection: SARS-CoV-2 and Ebola virus require cholesterol for viral entry (39–41), dengue virus requires triglycerides as an energy source through β-oxidation (42), and enteroviruses and flaviviruses require phosphatidylinositol-4-phosphate (PI4P) in the replication center for viral RNA replication (43).

In C. elegans the ortholog of human *srebp1* is *sbp-1*, and there is no ortholog of *srebp2* (44). *sbp-1* is involved in the regulation of lipogenic enzymes grouped in three main branches that generate the lipid precursors for the cell: the stearoyl-coenzyme A (CoA) desaturases *fat-6/fat-7*, the stearoyl-coenzyme A desaturase *fat-5*, and the monomethyl branched-chain fatty acids *elo-5/elo-6* (45). RNA interference knockdown of *sbp-1* or mutations in the *sbp-1* gene lead to animals with low fat stores, high saturated fatty acid content, and reduced expression of lipogenic genes (44, 46–48). Interestingly, mutation of *sbp-1* in C. elegans also leads to ~2-fold accumulation of zinc (49). A genetic suppressor screen identified that mutation in *sur-7*, which encodes a member of the cation diffusion facilitator family, reduces the accumulation of zinc in *sbp-1* mutants and also restores lipid levels (49), suggesting there is a link between zinc and lipid homeostasis. Only limited prior studies have investigated linkages between zinc and lipids. For example, zinc induces lipophagy in primary hepatocytes of yellow catfish (50), zinc levels are reduced in patients with alcoholic fatty liver disease (51), zinc supplementation reduces total cholesterol and triglycerides as found in a meta-analysis of 24 studies on humans, and zinc reverses alcoholic steatosis in mice (52). Related to virus infection, antiviral effects of zinc supplementation against multiple viruses, including herpesviruses (53), picornaviruses (54, 55), influenza virus (56), coronavirus (57), HCV (58–60), and HIV (61, 62), have been described. While different mechanisms have been proposed to explain the putative antiviral effect of zinc, including inhibition of viral protein cleavage and inhibition of viral polymerase activity (54, 55, 57), experimental data for these models are lacking, and it is not clear how zinc impacts virus infection.

Here, we show *in vivo* the impact of Orsay virus on lipid homeostasis as well as its dependence on lipid levels. We found that multiple transcription factors involved in lipid homeostasis play roles during Orsay virus infection, including the master regulator *srebp-1*/*sbp-1*. The reduced Orsay virus RNA levels in *sbp-1* mutant animals could be rescued biochemically by supplementation with specific lipids, genetically by introduction of the suppressor gene *sur-7*, and by pharmacological treatment with a chelator of zinc, TPEN [*N*,*N*,*N*′,*N*′-tetrakis(2-pyridylmethyl)ethane-1,2-diamine]. Finally, direct treatment of wild-type (WT) animals with zinc reduced Orsay virus RNA levels. In these studies, we were able to observe correlated changes in lipid abundance and Orsay virus RNA levels, demonstrating a connection between zinc, lipids, and virus infection.

## RESULTS

### Orsay virus infection reduced lipid levels in live animals.

**FIG 1:**
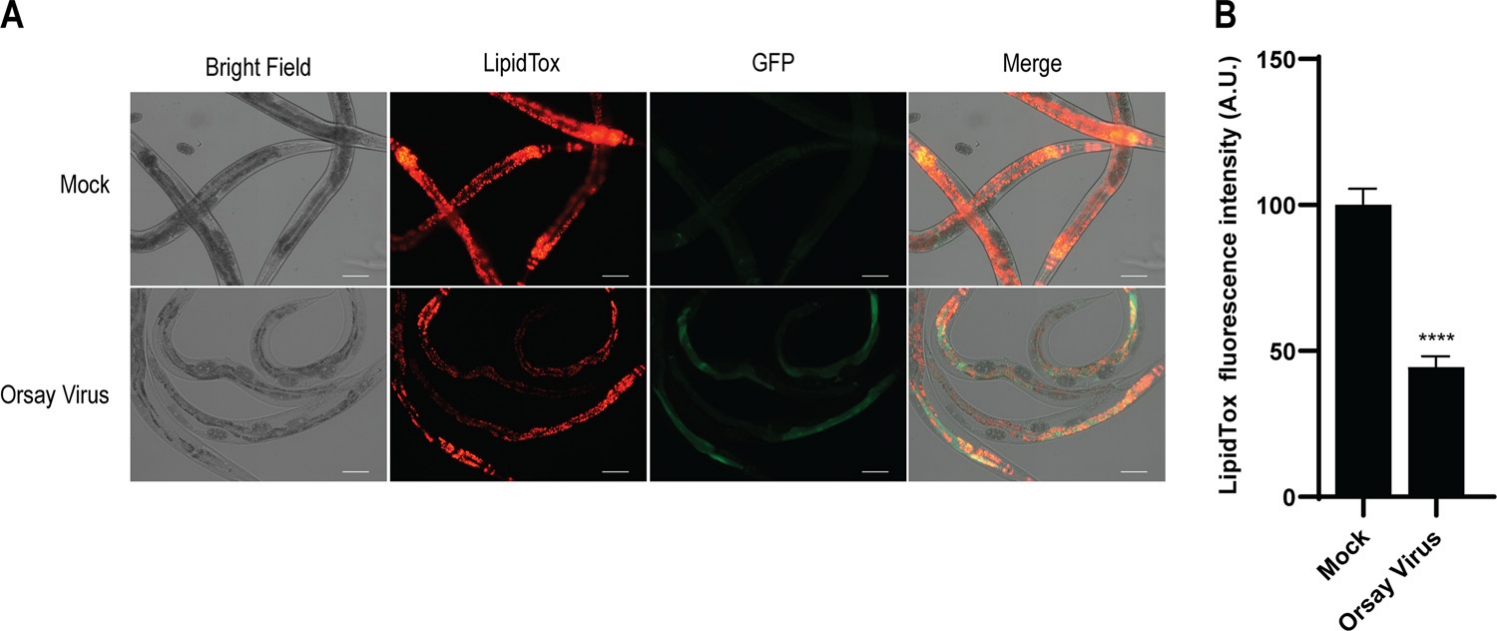
Orsay virus infection reduces lipid abundance in C. elegans. (A) The lipids from the *jyIs8*;*rde-1* strain, mock infected or infected with Orsay virus, were stained with LipidTox and visualized at 48 hpi. Mock represents noninfected animals, and GFP represents the green fluorescent protein of the reporter strain. The scale bar represents 100 μm. (B) Lipid levels were measured by quantifying the fluorescence intensity using an ArrayScan VTI HCS reader. The fluorescence intensity (shown in arbitrary units [A.U.]) was normalized by setting the value of the mock-infected sample as 100. Data are the arithmetic mean ± standard error of the mean from three independent experiments performed in duplicate. Statistically significant differences were determined by two-tailed *t* test. ****, *P* < 0.0001.

To examine the possibility that Orsay virus infection alters lipid levels in C. elegans, we quantified lipid abundance in live animals by lipid staining and microscopy. We employed a previously described transcriptional reporter strain, *jyls8;rde-1*(*ne219*), which is hypersensitive to Orsay virus and carries an integrated transcriptional green fluorescent protein (GFP) reporter driven by the *pals-5* promoter that is induced by Orsay virus and enables visualization of viral infection by fluorescence microscopy (6). Animals were fed the fluorescent dye LipidTox, which binds to neutral lipids such as triacylglycerols (63–65), and assayed at 48 h postinfection (hpi). Lipid abundance was significantly reduced by ~60% in animals that were infected with Orsay virus compared to noninfected control animals (Fig. 1). Similar reduction was observed following staining of animals with a different lipid staining dye, oil red O (ORO) (see Fig. S1 in the supplemental material). One possibility about this reduction is that lipids interfere with viral replication, and Orsay virus gets rid of lipids to help itself. In this case, reducing lipids will help the virus replicate. Another possibility is that lipids are necessary for replication and are depleted by viral activity. In this case, reducing lipids will blunt viral replication.

### Transcription factors that regulate lipid homeostasis are important for Orsay virus infection.

**FIG 2:**
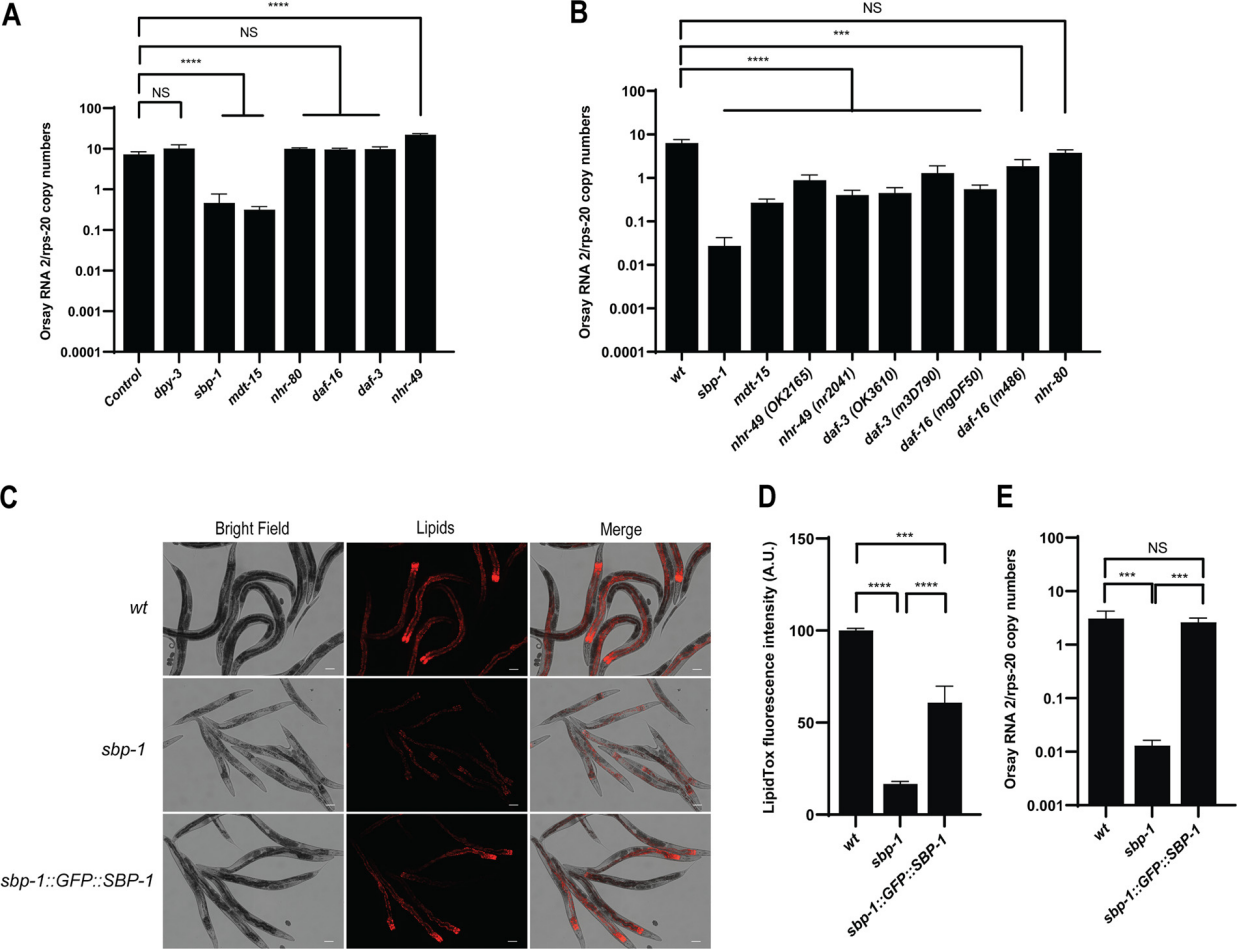
Transcription factors involved in lipid homeostasis play a role during Orsay virus infection. (A) RNAi knockdown of transcription factors in *drh-1* mutant animals. Animals were fed with RNAi bacteria targeting the indicated genes, and viral RNA levels were quantified by qRT-PCR. Control represents an empty vector without any targeted gene. (B) Mutant animals with mutations of the corresponding genes were challenged with Orsay virus, and the viral RNA levels were obtained by qRT-PCR. Data are the arithmetic mean ± standard error of the mean from at least three independent experiments performed in triplicate. Statistically significant differences were determined by Mann-Whitney test. ***, *P* = 0.001; ****, *P* < 0.0001. Values that were not statistically significantly different (*P* > 0.05) (NS) are indicated. (C) Fluorescence microscopy of lipid levels in *sbp-1*::*GFP*::*SBP-1* (CE548) strain. The scale bar represents 100 μm. (D) Quantification of data from panel C. The fluorescence intensity (shown in arbitrary units [A.U.]) was normalized by setting the value of WT as 100. Data are the arithmetic mean ± standard error of the mean of three independent experiments performed in duplicate. Statistically significant differences were determined by one-way ANOVA with a statistical difference identified between three *post hoc* comparisons analyzed by Fisher’s multiple-comparison test (***, *P* = 0.001; ****, *P* < 0.0001). (E) Wild-type (wt), mutant *sbp-1*(*ep79*), and *sbp-1*::*GFP*::*SBP-1* (CE548) animals were challenged with Orsay virus, and the viral RNA levels were quantified by real-time qRT-PCR. Data are the arithmetic mean ± standard error of the mean from three independent experiments performed in triplicate. Statistically significant differences were determined by Kruskal-Wallis test with a statistical difference identified between three *post hoc* comparisons analyzed by Dunn’s multiple-comparison test (***, *P* < 0.007; NS, not significant [*P* > 0.05]).

To determine whether Orsay virus depends on lipids for infection, we quantified Orsay virus RNA levels in animals depleted of lipids. First, we used RNA interference to knock down the major transcription factors, *sbp-1*, *nhr-80*, *nhr-49*, *daf-3*, and *daf-16*, as well as the transcriptional mediator *mdt-15*, known to be involved in the lipid biosynthesis of C. elegans (66). RNAi knockdown of *sbp-1* and *mdt-15* reduced the Orsay virus RNA levels by ~14- and ~21-fold, respectively, at 48 hpi compared to the control, empty RNAi feeding vector or knockdown of an irrelevant gene, *dpy-3* (Fig. 2A). To corroborate this finding, we tested animals carrying defined mutations in these genes that were available from the Caenorhabditis Genetics Center (CGC). We demonstrated that mutants in *nhr-49*(*ok2165* and *nr2041*), *daf-3*(*ok3610* and *m3D790*), *daf-16*(*m486* and *mgDF50*), and *mdt-15*(*tm2182*) displayed viral RNA levels that were reduced ~16-, ~14.5-, ~11.6-, and ~23.6-fold, respectively (Fig. 2B). Of all the mutants we tested, only *nhr-80*(*tm1011*) did not display reduced viral RNA levels. Interestingly, *sbp-1*(*ep79*), which has a deletion of 2.1 kb resulting in partial loss of function (67), caused the strongest phenotype with an ~236-fold reduction of Orsay virus viral RNA (Fig. 2B). These results suggest that Orsay virus requires lipids for an efficient infection and that the evolutionarily conserved master regulator *sbp-1*(*ep79*) is playing a major role during Orsay virus infection in C. elegans.

To unambiguously demonstrate that the viral phenotype in the *sbp-1*(*ep79*) mutant is due to SBP-1, we evaluated Orsay virus infection in a transgenic *sbp-1*(*ep79*) strain that overexpresses SBP-1 fused to GFP (*sbp-1*::GFP::SBP-1, CE548) (68). First, we evaluated the recovery of lipid production in the *sbp-1*::GFP::SBP-1 strain; for this, we performed lipid staining and found that the mutation increased lipid levels ~3.7-fold compared to *sbp-1*(*ep79*) (Fig. 2C and D). Likewise, when we challenged the *sbp-1*::GFP::SBP-1 strain with Orsay virus, it displayed viral RNA levels restored to those of wild-type animals (Fig. 2E). This result demonstrates the specific role of *sbp-1* and reinforces the idea that lipids promote Orsay virus infection in C. elegans.

### Genetic analysis of the *sbp-1* pathway identified a role for the *fat-6/fat-7* and *elo-5/elo-6* branches during Orsay virus infection.

**FIG 3:**
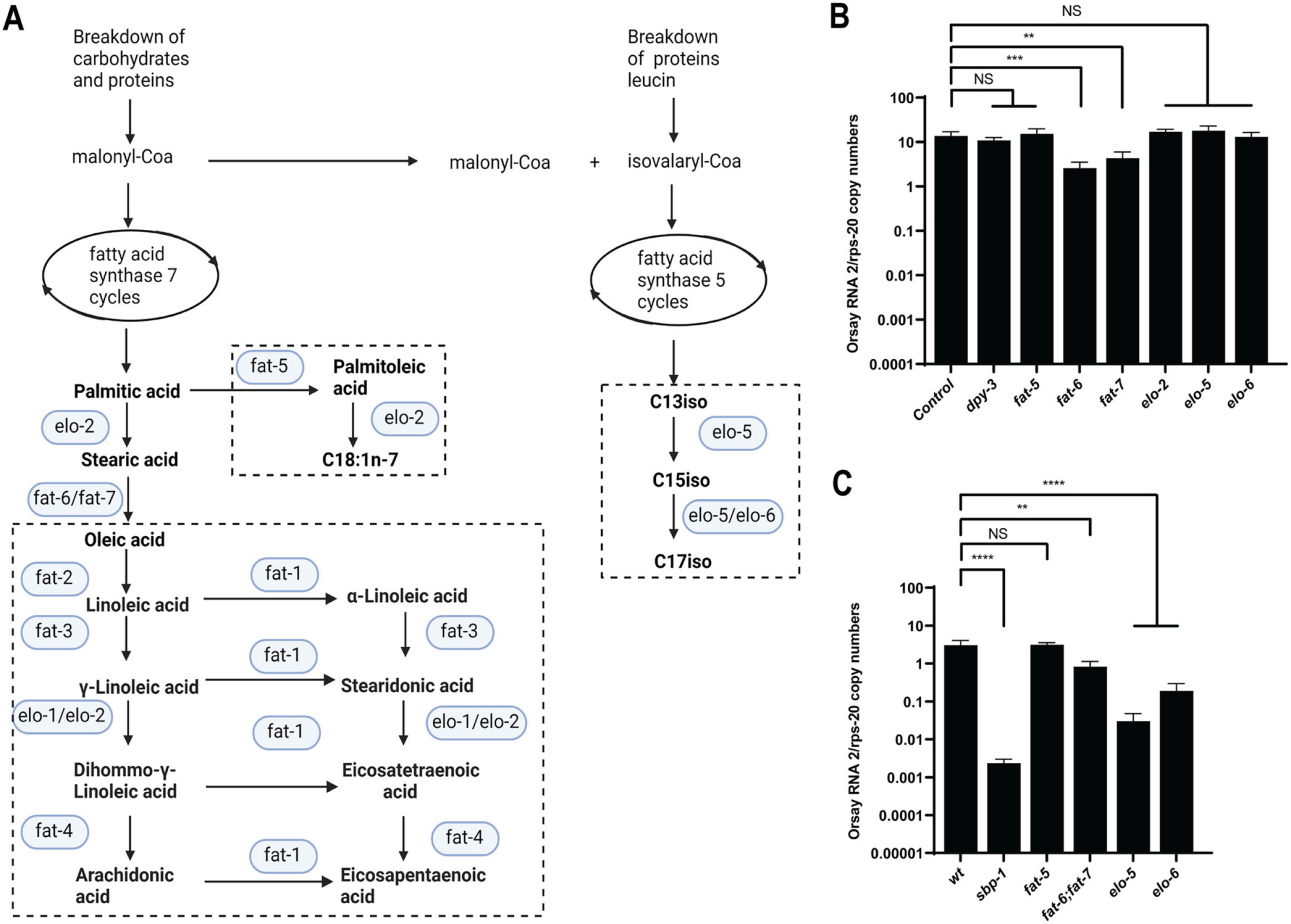
*fat-6/fat-7*, *elo-5*, and *elo-6* are important for Orsay virus infection. (A) A schematic representation of the three lipid branches regulated by *sbp-1*: *fat-6/fat-7*, *fat-5*, and *elo-5* (adapted from reference 45, 107). (B) Animals were fed with RNAi bacteria targeting the indicated genes, and viral RNA levels were quantified by real-time qRT-PCR. (C) Wild-type and mutant animals with mutations of the corresponding genes were challenged with Orsay virus, and the viral RNA levels were quantified by real-time qRT-PCR. Data are the arithmetic mean ± standard error of the mean from at least three independent experiments performed in triplicate. Statistically significant differences were determined by Mann-Whitney test. **, *P* < 0.005; ***, *P* = 0.0008; ****, *P* < 0.0001; NS, not significant (*P* > 0.05).

*sbp-1* is a transcriptional activator of the expression of multiple lipogenic enzymes in three different branches (Fig. 3A) (30, 45). To identify which branch(es) regulated by *sbp-1* is important for Orsay virus infection, we performed an RNA interference-mediated knockdown of representative lipogenic genes from every branch (Fig. 3A and B). Knockdown of *fat-6* and *fat-7*, which encode stearoyl-coenzyme A (CoA) desaturases and which function redundantly to synthesize oleic acid from stearic acid, reduced the Orsay virus RNA levels; however, knockdown of *elo-2*, which is involved in the synthesis of stearic acid, or the pathways represented by *fat-5* or *elo-5* and *elo-6* did not alter Orsay virus RNA levels.

As the levels of knockdown by RNAi are likely to be incomplete (69), we challenged mutant animals that lacked these same genes with Orsay virus, except for *elo-2*, which is an essential gene (70). The *fat-6*(*tm331*);*fat-7*(*wa36*) double mutant animals, which are deficient in oleic acid production (71, 72), had ~5-fold-reduced Orsay virus RNA levels. No phenotype was observed for the *fat-5*(*tm420*) mutant animals, which are deficient in the production of palmitoleic acid (72), which suggests that the lipids from this branch are not critical for Orsay virus infection. Interestingly, in the *elo-5*(*gk182*) and *elo-6*(*gk233*) mutant animals Orsay virus RNA levels were reduced by ~65-fold and ~10-fold, respectively (Fig. 3C), in contrast to the RNAi results in which gene expression was knocked down but not ablated.

### A biochemical screen of the *sbp-1* pathway identified lipids that rescue Orsay virus infection.

**FIG 4:**
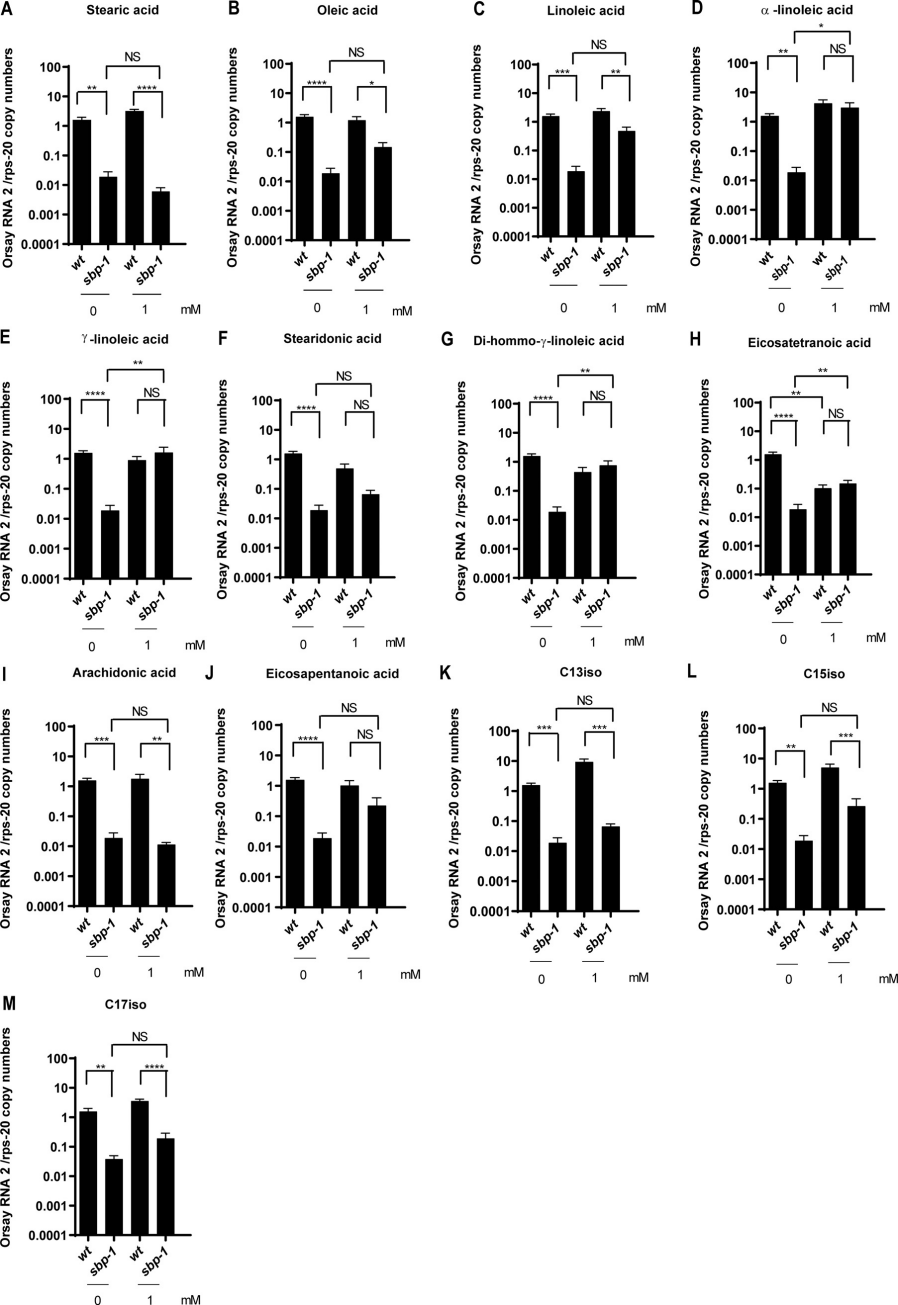
(A) Stearic acid, (B) Oleic acid, (C) Linoleic acid, (D) α-linoleic acid, (D) γ-linoleic acid, (E) Stearidonic acid, (F) Stearidonic acid, (G) Di-hommo-γ-linoleic, (H) Eicosatetranoic acid, (I) Arachidonic acid, (J) Eicosapentanoic acid, (K) C13iso, (L) C15iso, M (C17iso). RNA levels were quantified by real-time qRT-PCR. Data are the arithmetic mean ± standard error of the mean from three independent experiments performed in triplicate. Statistically significant differences were determined by Kruskal-Wallis test with a statistical difference identified between four *post hoc* comparisons analyzed by Dunn’s multiple-comparison test (*, *P* < 0.0311; **, *P* < 0.0089; ***, *P* < 0.0005; ****, *P* < 0.0001). NS, not significant (*P* > 0.05).

In the *sbp-1*(*ep79*) mutant, there is accumulation of the saturated fatty acid stearic acid, whereas several lipid products like oleic acid and linoleic acid, or monomethyl branched-chain fatty acids like C15iso and C17iso are reduced (48, 67). To determine which lipid(s) was limiting in the *sbp-1*(*ep79*) mutant for Orsay virus infection, we biochemically supplemented the animals with a range of lipids. After 3 days postinfection (dpi), the animals were collected, and the viral RNA was quantified by quantitative reverse transcription-PCR (qRT-PCR). As expected, feeding with stearic acid had no impact on *sbp-1*(*ep79*) animals, as stearic acid is upstream of the genes regulated by *sbp-1*. Likewise, we did not find rescue of the viral RNA levels for animals fed with oleic acid or linoleic acid (Fig. 4B and C). The lipids α-linoleic acid, γ-linoleic acid, and dihomo-γ-linoleic acid completely rescued the viral RNA levels to wild-type (WT) levels (Fig. 4D, E, and G). However, supplementation with lipids like eicosatetraenoic acid had complex effects and also reduced the viral RNA levels in WT animals (Fig. 4H). Furthermore, lipid supplementation with arachidonic or eicosapentaenoic acid did not rescue the viral RNA levels (Fig. 4I and J). In addition, the lipids C15iso and C17iso from the *elo-5/elo-6* branch did not rescue the viral RNA levels (Fig. 4K to M). These results demonstrate that supplementation with specific lipids in the *sbp-1* background can rescue Orsay virus infection.

### *sbp-1* functions at the replication stage of the Orsay virus life cycle.

**FIG 5:**
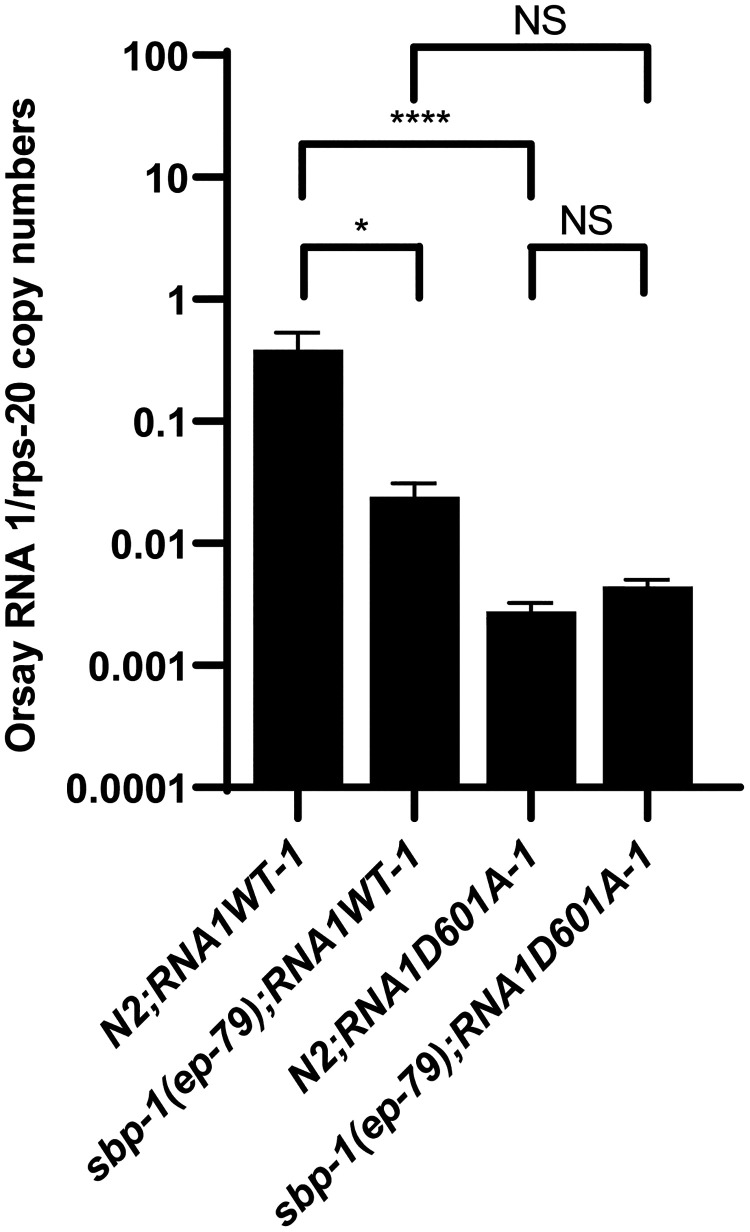
An *in vivo* replicon system displayed that Orsay virus replication is reduced in *sbp-1*(*ep79*) mutant animals. Strains harboring RNA1 alone are competent to support replication of the Orsay virus RNA1 segment following heat shock induction. Quantification of Orsay virus RNA1 replication from heat-induced transgenic C. elegans was performed by qRT-PCR. Two independent replicon lines were generated in the wild-type (N2) and mutant *sbp-1*(*ep79*) animals (see Fig. S1 in the supplemental material). As a negative control for replication, two independent lines with a defective polymerase (DP) were generated in N2 and *sbp-1*(*ep79*) mutant animals (see Fig. S1 in the supplemental material). Data are the arithmetic mean ± standard error of the mean from three independent experiments performed in triplicate. Statistically significant differences were determined by Kruskal-Wallis test with a statistical difference identified between four *post hoc* comparisons analyzed by Dunn’s multiple-comparison test (*, *P* < 0.0396; ****, *P* < 0.0001). NS, not significant (*P* > 0.05).

Because the depletion of lipids can impact many stages of the viral life cycle, we sought to determine whether *sbp-1*(*ep79*) mutation was directly affecting the viral replication step of the Orsay virus life cycle. We employed a previously described replicon system for Orsay virus based on an extrachromosomal array of plasmids of the entire Orsay virus WT RNA1 segment that encodes a WT RNA-dependent RNA viral polymerase (RdRp) (WUM104 [N2;PHIP::RNA1WT]) under a heat shock promoter (6, 9, 13). Strains harboring RNA1 alone are competent to support replication of the Orsay virus RNA1 segment following heat shock induction. Also, as a control to determine the level of Orsay virus RNA generated by DNA-templated transcription, a transgenic wild-type strain carrying extrachromosomal arrays of a D601A polymerase-dead mutant of Orsay virus RNA1 (WUM106 [N2;PHIP::RNA1D601A]) was also generated. These transgenic strains were crossed with the *sbp-1*(*ep79*) mutant to generate the replicon system in this background and compare the viral RNA levels of this strain with those of wild-type animals. The replicon system was induced by heat shock, and the Orsay virus RNA levels were quantified by qRT-PCR. In the *sbp-1*(*ep79*) mutant carrying the RNA1 WT replicon {WUM108 [*sbp-1*(*ep79*);PHIP::RNA1WT]}, there is no statistical difference from WUM110 [*sbp-1*(*ep79*);*PHIP*::*RNA1D601A*], and these viral RNA levels were ~55-fold lower than those of the N2;PHIP::RNA1WT animals (Fig. 5 and Fig. S2). These results suggest that *sbp-1* is necessary for Orsay virus replication. This analysis does not rule out the possibility that *sbp-1* may also act at other stages of the Orsay virus life cycle.

### Zinc impacts lipid levels and Orsay virus infection in wild-type and *sbp-1*(*ep79*) mutant animals.

**FIG 6:**
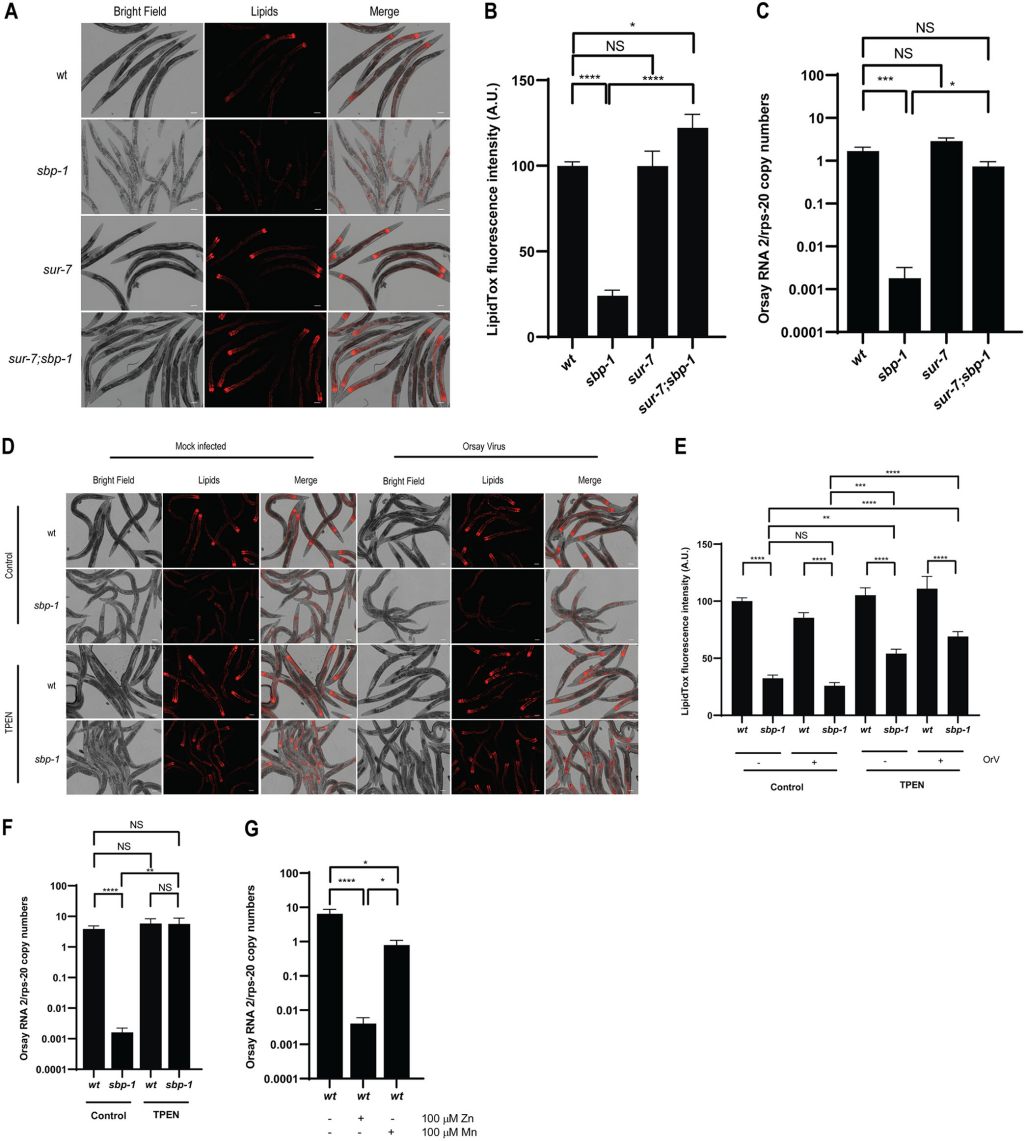
Zinc levels impact Orsay virus infection in N2 and *sbp-1*(*ep79*). (A) The lipid levels of the wild-type, *sbp-1*(*ep79*), *sur-7*(*ku119*), and double mutant *sur-7*;*sbp-1*(*ep79*) animals were analyzed by staining and microscopy. The scale bar represents 100 μm. (B) The fluorescence intensity (shown in arbitrary units [A.U.]) was normalized by setting the value of WT as 100. Statistically significant differences were determined by one-way ANOVA with a statistical difference identified between four *post hoc* comparisons analyzed by Fisher’s multiple-comparison test (*, *P* = 0.0201; ****, *P* < 0.0001; NS, not significant [*P* > 0.05]). (C) Wild-type, *sbp-1*(*ep79*), *sur-7*(*ku119*), and double mutant *sur-7*;*sbp-1*(*ep79*) animals were challenged with Orsay virus, and the viral RNA levels were quantified by qRT-PCR. Data are the arithmetic mean ± standard error of the mean from three independent experiments performed in triplicate. Statistically significant differences were determined by Kruskal-Wallis test with a statistical difference identified between four *post hoc* comparisons analyzed by Dunn’s multiple-comparison test (*, *P* = 0.0157; ***, *P* = 0.0002; NS, not significant [*P* > 0.05]). (D) Imaging of lipids of the animals treated with TPEN. “Mock infected” represents animals not infected with Orsay virus. The scale bar represents 100 μm. (E) Lipid staining and quantification of data from panel D. The fluorescence intensity (shown in arbitrary units [A.U.]) was normalized by setting the value of WT in the control dish as 100. Data are the arithmetic mean ± standard error of the mean from three independent experiments performed in duplicate. Statistically significant differences were determined by one-way ANOVA with a statistical difference identified between nine *post hoc* comparisons analyzed by Fisher’s multiple-comparison test (**, *P* = 0.0074; ***, *P* = 0.0007; ****, *P* < 0.0001; NS, not significant [*P* > 0.05]). (F) Chelation of zinc with 1 μM TPEN in wild type and *sbp-1*(*ep79*) mutants was performed, and the viral RNA levels were measured. Statistically significant differences were determined by Kruskal-Wallis test with a statistical difference identified between four *post hoc* comparisons analyzed by Dunn’s multiple-comparison test (**, *P* = 0.0022; ****, *P* < 0.0001; NS, not significant [*P* > 0.05]). (G) Wild-type animals cultured with 100 μM zinc or 100 μM manganese were challenged with Orsay virus, and the viral RNA levels were quantified by qRT-PCR. Data are the arithmetic mean ± standard error of the mean from three independent experiments in triplicate. Statistically significant differences were determined by Kruskal-Wallis test with a statistical difference identified between three *post hoc* comparisons analyzed by Dunn’s multiple-comparison test (*, *P* < 0.0302; ****, *P* < 0.0001).

The observation that specific lipids can rescue Orsay virus infection in the *sbp-1*(*ep79*) mutant demonstrated that lipid deficiency of the *sbp-1* mutant is important. However, the factors involved in lipid homeostasis are still not clear. Interestingly, a forward genetic screen identified *sur-7* as a suppressor of the *sbp-1*(*ep79*) mutant that restores lipid levels (49). *sur-7* encodes a member of the cation diffusion facilitator family and is involved in zinc metabolism (73, 74). To investigate if the mutation in *sur-7*(*ku119*) could rescue the lipid levels in the *sbp-1*(*ep79*) mutant, we performed LipidTox staining. We confirmed that the *sur-7*(*ku119*);*sbp-1*(*ep79*) double mutant rescued lipid levels to those of wild-type animals (Fig. 6A and B). Staining with ORO also demonstrated a significant increase in lipid levels in the *sur-7*(*ku119*);*sbp-1*(*ep79*) background compared to *sbp-1*(*ep79*) (Fig. S3). Likewise, to investigate if the virus infection in the *sbp-1*(*ep79*) mutant could be rescued, we challenged the *sur-7*(*ku119*);*sbp-1*(*ep79*) double mutant animals with Orsay virus. Orsay virus RNA levels in the double mutant were increased compared to the *sbp-1*(*ep79*) mutant and similar to those of wild-type animals (Fig. 6C).

**FIG 7:**
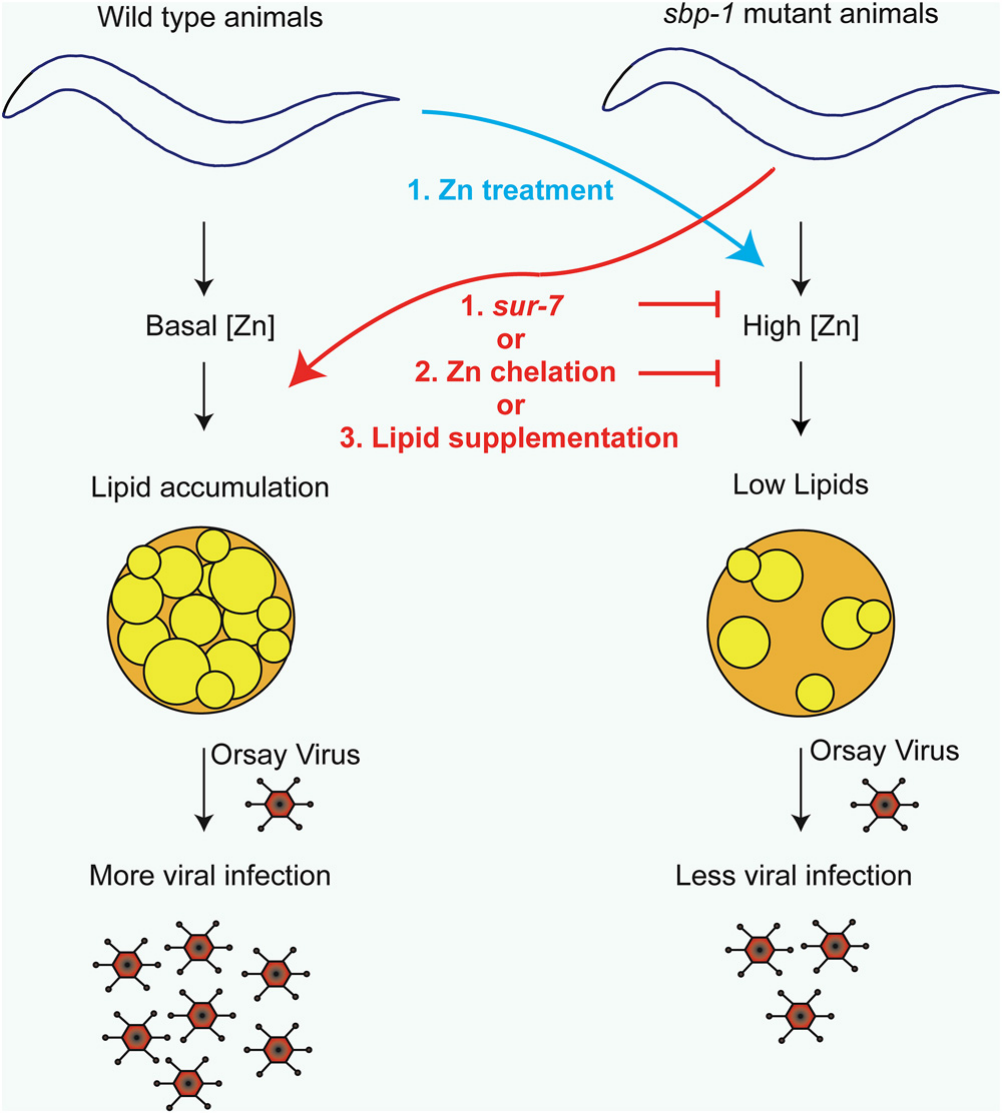
Zinc-lipid interaction upon C. elegans infection. In wild-type animals there are basal levels of zinc that allow the proper accumulation of lipids. Upon infection, Orsay virus utilizes host lipids for efficient replication and production of viral progeny. In *sbp-1*(*ep79*) mutant animals, there is a high concentration of zinc and lower levels of lipids, and it results in less virus infection. Mutation of *sur-7* or chelation of zinc by TPEN in *sbp-1*(*ep79*) mutant animals lowers zinc concentration and rescues the lipid levels, which rescue the Orsay virus infection. Likewise, specific lipid supplementation with α-linoleic acid, γ-linoleic acid, and dihomo-γ-linoleic acid of the *sbp-1*(*ep79*) animals is sufficient to rescue the Orsay virus infection.

Because it is known that mutation of *sbp-1* in C. elegans leads to accumulation of zinc, and *sur-7* suppresses both the increased zinc and decreased lipid phenotypes in the *sbp-1* mutant (49), we used a pharmacological strategy to specifically deplete the zinc levels in the *sbp-1*(*ep79*) mutant. If a high concentration of zinc is depleting the lipid levels in the *sbp-1*(*ep79*) mutant and this is reducing the Orsay virus infection, then reduction of zinc levels in the *sbp-1*(*ep79*) mutant should rescue the lipid production and rescue the virus infection. If zinc is not important for lipid homeostasis, then zinc depletion should not rescue the lipid levels and the Orsay virus infection. Addition of TPEN, a membrane-permeant zinc chelator (49, 75), to the medium increased the lipid levels in the *sbp-1*(*ep79*) mutant up to ~2.6-fold (Fig. 6D and E). Likewise, TPEN treatment rescued the Orsay virus RNA levels in the *sbp-1*(*ep79*) mutant to levels similar to those in wild-type animals (Fig. 6F). This result suggests that high concentrations of zinc in the *sbp-1*(*ep79*) mutant play a role in the reduction of the lipid levels, thereby preventing efficient Orsay virus infection (hypothetical model shown in Fig. 7). Thus, Orsay virus infection of the *sbp-1* mutant could be rescued by multiple approaches: (i) ectopic supplementation of various lipids, (ii) mutation of the *sur-7* gene, and (iii) pharmacological depletion of zinc with TPEN.

Since reducing zinc levels helps to recover the Orsay virus infection, this suggests that increasing the zinc concentration might hurt the virus. We tested this prediction and supplemented zinc into the medium of wild-type N2 animals. Supplementation of zinc in wild-type animals reduced the viral RNA levels ~1,620-fold compared to their respective controls in standard medium (Fig. 6G). Treatment with a different divalent cation, manganese (Mn), reduced the viral RNA levels by only ~8-fold in the wild-type animals, demonstrating a marked impact of zinc (hypothetical model in Fig. 7).

## DISCUSSION

The genetic tractability of C. elegans and its conservation of many pathways with mammals make it an excellent reductionist model to explore host-virus interactions. Use of Orsay virus enables the study of host-virus interactions *in vivo* in a natural multicellular host. Here, we explored the role of lipids in Orsay virus infection. We demonstrate that Orsay virus impacts lipid homeostasis by reducing the amount of lipids ~60% by 48 hpi in animals, suggesting that Orsay virus alters lipid metabolism during viral infection. Interestingly, in human cells, dengue virus similarly reduces lipid abundance by 60% at 24 to 48 hpi (42).

The dependence of Orsay virus on lipids is also supported by assessing the impact of genetic approaches to depleting lipids. Animals with mutations of multiple transcription factors as well as RNAi knockdown of those transcription factors had reduced Orsay virus RNA levels. Although by RNAi we observed phenotypes for only *sbp-1* and *mdt-15*, defined mutants of additional transcription factors also led to lower Orsay virus RNA levels, most likely due to incomplete knockdown by RNAi. The observed dependence of Orsay virus in C. elegans on lipids parallels the observation that depletion of lipids reduces infection by many pathogenic mammalian viruses, including SARS-CoV-2 (76), hepatitis C virus (77), Zika virus (78), poliovirus (PV) (79), and encephalomyocarditis virus (EMCV) (80). Thus, Orsay virus infection of C. elegans may serve as a robust model of these interactions.

As *sbp-1* is involved in the expression of many lipogenic genes, we sought to determine which lipid biosynthetic genes and corresponding lipid molecules are required during infection. We found genetically that of the three branches regulated by *sbp-1*, the *fat-6/fat-7* and *elo-5/elo-6* branches affected Orsay virus infection but the *fat-5* pathway did not. *sbp-1*(*ep79*) mutants accumulate fatty acids like palmitic and palmitoleic acid, which belong to the *fat-5* branch, but have reduced levels of oleic acid, linoleic acid, and C15iso/C17iso, which correspond to the *fat-6/fat-7* and *elo-5/elo-6* branches, respectively (48, 67). Feeding the *sbp-1*(*ep79*) mutants biochemically with multiple lipids from the *fat-6/fat-7* branch, including α-linoleic acid and γ-linoleic acid and dihomo-γ-linoleic acid, rescued viral RNA levels, but stearic acid, arachidonic acid, C15iso, or C17iso did not. The inability of stearic acid to rescue is predicted since stearic acid is the precursor of the *fat-6/fat-7* enzymes and the *sbp-1*(*ep79*) mutant accumulates stearic acid. The observation that multiple lipids downstream of *fat-6/fat-7* rescued Orsay virus RNA levels confirms the importance of this branch of lipid metabolism for Orsay virus. We did not observe rescue of the *sbp-1*(*ep79*) mutant by supplementation of C15iso or C17iso even though mutants with mutations in *elo-5* and *elo-6*, the genes that synthesize these two lipids, respectively, have lower levels of Orsay virus infection. One possible explanation is that in the *sbp-1*(*ep79*) mutant there is a reported 50% reduction in the C15iso/C17iso lipid levels whereas in *elo-5* and *elo-6* mutants, which have a deletion of 379 bp and 184 bp of the first exon, respectively, the reduction is likely to be greater; therefore, the levels of these two lipids may not be limiting factors in the *sbp-1* background (67).

Lipids could be required for one or more steps of the Orsay virus life cycle, such as viral entry, proper release and trafficking of infectious particles into the cytoplasm, replication of viral RNA, viral assembly, or egress. To better define the stage at which *sbp-1* acts, we specifically assessed viral replication by employing an *in vivo* replicon system where Orsay virus replication is initiated from an inducible integrated transgene (6). We found that virus RNA levels were reduced in the *sbp-1*(*ep79*) mutant, indicating that one or more lipids regulated by *sbp-1* are needed for virus RNA replication. While a number of studies have defined the importance of *srebp-1*, the human ortholog of *sbp-1*, for viral infection, none of the studies have implicated a specific stage of the viral life cycle that is dependent on *srebp1* (32, 35, 37, 81). Thus, our findings demonstrate that the step of viral RNA replication is affected in the *sbp-1*(*ep79*) mutant, although it is possible that additional other steps of the viral life cycle may be also affected.

These findings demonstrate that specific lipids regulated by *sbp-1* are important for Orsay virus infection. The exact mechanism by which these lipids are necessary for an efficient virus replication is still unknown, but several possibilities center on roles involving the viral replication center. It has been shown that oleic acid plays a role in the viral replication step of hepatitis C virus (HCV), as its depletion disrupts the integrity of membranous HCV replication centers and renders HCV RNA susceptible to nuclease-mediated degradation (82). Another possibility is that the lipids could be the precursors for components of the replication center that help to recruit the RdRp. Such is the case for coxsackievirus B3 (CVB3) and poliovirus (PV) RNA polymerases that show a high affinity for PI4P lipids (43) or the p92 RNA polymerase from tomato bushy stunt virus (TBSV), which recognizes phosphatidylethanolamine (PE) to form a complex associated with the membrane of peroxisomes (83). Alternatively, or in addition, lipids can directly modulate viral enzymatic activities. For example, the autocatalytic cleavage of the PV 3CDpol proteins, which are the precursors of the polymerase (3Dpol) and protease 3C, is attenuated when bound to PI4P lipids (84). Likewise, it has been shown that PE stimulates the enzymatic activity of TBSV viral polymerase and enhances its association with viral RNA (83), whereas binding to phosphatidylglycerol lipids inhibits its activity (85). The ability of some lipids, but not others, to rescue Orsay virus infection in C. elegans provides an opportunity to further dissect the precise biochemical interactions required for virus infection. More studies of the localization of the critical lipids, as well as their downstream products, are necessary to understand how Orsay virus affects and is dependent upon lipid metabolism *in vi*vo.

One strength of the C. elegans system is its genetic tractability, which enables detailed dissection of pathways by genetic approaches including suppressor screens. A previous suppressor screen of the *sbp-1*(*ep79*) mutant lipid defect found that mutation of *sur-7*, which encodes a transporter of zinc, restores lipid homeostasis (49). We found that mutation of *sur-7* rescued the lipid levels as well as the Orsay virus infection in the *sbp-1* background. These results suggested that zinc might be playing a role in the homeostasis of lipids and thus affecting the virus infection. In concordance with this hypothesis, zinc chelation in the *sbp-1* background rescued both lipid levels and Orsay virus infection. It is only due to the unbiased nature of the forward genetic suppressor screening that this linkage between zinc, lipid homeostasis, and virus infection was hypothesized. Interestingly, other papers have shown a correlation between zinc and lipids (50, 86), and antiviral effects of zinc supplementation against multiple mammalian viruses have been described (53–55, 57, 61, 87, 88). Although some mechanisms have been proposed to explain the role of zinc during virus infection, like the inhibition of viral protein cleavage and processing as well as inhibition of the viral polymerase activity, the experimental data supporting these mechanistic models are lacking (54, 55, 57). Interestingly, the importance of lipids for many of these zinc-sensitive viruses including coronaviruses (26, 89), picornaviruses (43, 90), and hepatitis E virus (91), has been reported. Our collective data support a novel hypothetical mechanistic model wherein zinc reduces virus infection via the depletion of lipids and may have broad implications for zinc-sensitive viruses (Fig. 7). In addition, this reaffirms the great potential of model organism studies to elucidate novel mechanisms and dissect pathways that are broadly important across host species.

## MATERIALS AND METHODS

### C. elegans culture and maintenance.

C. elegans N2 (wild-type), CE548 (SBP-1:GFP), *drh-1*(*ok3495*), *sbp-1*(*ep79*), *nhr-49*(*ok2165* and *nr2041*), *daf-3*(*ok3610* and *mgDf90*), *daf-16*(*mu86* and *mgDf50*), *mdt-15*(*tm2182*), *nhr-80*(*tm1011*), *fat-5*(*tm420*), *fat-6*(*tm331*);*fat-7*(*wa36*), *elo-5*(*gk182*), *elo-6*(*gk233*), and *sur-7*(*ku119*) strains were obtained from the Caenorhabditis Genetics Center (CGC); these strains were maintained under standard lab culture conditions unless otherwise specified. In brief, animals were fed Escherichia coli OP50 on nematode growth medium (NGM) dishes in a 20°C incubator and moved every 3 days to a new NGM dish seeded with E. coli OP50 (92).

### Orsay virus preparation, infection, and RNA extraction.

Orsay virus was prepared by liquid culture as described previously (11), filtered through a 0.22-μm filter, and stored at −80°C. For all infection experiments, animals were bleached and then synchronized in M9 buffer (1) in 15-mL conical tubes with constant rotation at room temperature for 18 h. In a six-well dish with E. coli OP50, 500 arrested larval-stage 1 (L1) larvae were seeded. L1 larvae were allowed to recover for 20 h at 20°C prior to infection. Orsay virus filtrate was thawed at room temperature and then diluted 1:10 with M9 buffer. For each well, 20 μL of 2.5 × 10^5^ tissue culture infective doses (TCID_50_)/mL (multiplicity of infection [MOI] of 10) of virus filtrate was added into the middle of the bacterial lawn and incubated at 20°C. Three days after infection, animals were collected into 1.5-mL Eppendorf tubes by washing each well with 1 mL of M9 buffer and then pelleted by spinning for 1 min at 376 × *g* in a benchtop centrifuge. M9 supernatant was removed, 300 μL TRIzol reagent (Invitrogen) was added to the tubes, and then the tubes were frozen in liquid nitrogen. For each experiment, three replicate wells were used for each infection condition unless otherwise indicated. Total RNA from infected animals was extracted using Direct-zol RNA miniprep (Zymo Research) purification according to the manufacturer’s protocol and eluted into 30 μL of RNase/DNase-free water.

### *In vivo* lipid staining.

LipidTox Red Neutral lipid (1,000×; ThermoFisher H34476) was diluted in E. coli OP50 and dispensed on NGM agar dishes to yield a final concentration of 10×. A synchronized population of the *jyls8*;*rde-1*(*ne219*) strain (WUM31), in stage L1, was transferred to the dish and incubated for 20 h. Then the animals were mock infected or infected with 200 μL of a 1:10 dilution of Orsay virus in M9 buffer and collected at 48 h postinfection. Twenty-five animals were transferred to 384-well plates containing 60 μL of phosphate-buffered saline (PBS) and anesthetized with 25 mM tetramisole in M9. Total fluorescence intensity was acquired with the ArrayScan V HCS reader (Cellomics, ThermoFisher). Three independent experiments were performed in duplicate, making a total of 150 animals quantified per condition. Statistical testing was done by the parametric two-tailed *t* test or one-way analysis of variance (ANOVA) to compare two or more than two different groups, respectively.

### C. elegans RNAi feeding for knockdown.

RNAi feeding was used for gene knockdown as described previously (93). E. coli strain HT115, carrying double-stranded RNA expression cassettes for genes of interest, was induced using established conditions and then seeded into a six-well NGM dish. *dpy-3*, *sbp-1*, *mdt-15*, *nhr-49*, *daf-3*, *daf-16*, *nhr-80*, *fat-5*, *fat-6*, *fat-7*, *elo-2*, *elo-5*, and *elo-6* RNAi clones were from the Ahringer RNAi library (94). Twenty arrested L1 *drh-1* mutant animals were seeded into each well of a six-well dish. After 72 h of RNAi feeding, Orsay virus was added to the dishes as described above. At 48 h postinfection, the C. elegans animals were collected, and 300 μL of TRIzol (Invitrogen) was added for RNA extraction.

### Orsay virus quantification by real-time qRT-PCR.

RNA extracted from infected animals was subjected to one-step real-time quantitative reverse transcription-PCR (qRT-PCR) to quantify Orsay virus RNA as previously described (13). Briefly, the extracted viral RNA was diluted 1:100, and 5 μL was used in a TaqMan fast virus one-step qRT-PCR with primers and probe (OrV_RNA2) that target the Orsay virus RNA2 segment. Absolute Orsay virus RNA2 copy number was determined by comparison to a standard curve generated using serial dilutions of Orsay virus RNA2 *in vitro* transcripts. Primers GW314 and GW315 and probe OrV_RNA1, which target the Orsay virus RNA1 segment, were used to quantify Orsay virus RNA1 abundance (13). To control for variation in the number of animals, Orsay virus RNA levels were normalized to an internal control gene, *rps-20*, which encodes a small ribosomal subunit S20 protein required for translation in C. elegans (95). At least three independent experiments in triplicate were performed. Statistical testing was done employing the Mann-Whitney test to compare two different groups of samples when indicated or the Kruskal-Wallis test to compare samples in Fig. 2C, Fig. 4, Fig. 5, Fig. 6A, D, and G, and Fig. S1 in the supplemental material. Graphic representation and statistical analyses were performed using GraphPad Prism 9 software.

### Microinjection of the replicon system and analysis of the viral life cycle.

PHIP::RNA1 and PHIP::RNA1D601A mutant constructs, which encode the wild type (RNA1 WT) and dead viral polymerase (RNA1 DP), were microinjected into the gonad of animals to generate stable transgenic lines (6, 13). Briefly, 5 ng/μL of the constructs was mixed with 100 ng/μL of the transgenic marker Pmyo-3::YFP (yellow fluorescent protein) and 5 ng/μL of a 2log ladder (New England Biolabs [NEB]). C. elegans N2 young adults were injected by using a microinjection system (Zeiss). Sets of three microinjected animals were placed on E. coli OP50-seeded 6-well NGM dishes and maintained at 20°C. F1 animals displaying the fluorescent marker at the pharynx were individually transferred onto new E. coli OP50-seeded 6-well NGM dishes 3 to 5 days after injection. The stable transgenic array containing F2 animals was selected and maintained by picking 5 transgenic progeny animals each time onto a new E. coli OP50-seeded 6-well NGM dish (Table 1).

**TABLE 1:** Transgenic C. elegans strains used in this study

Lab designation	Strain name	Relevant genotype	Reference
WUM31	jyIs8;rde-1(ne219)	{jyIs8[Ppals-5::GFP;Pmyo-2::mCherry]; rde-1(ne219) V}	98
WUM104	N2;virEx64[PHIP::RNA1WT-1]	{virEx64[PHIP::OrsayRNA1WT-1; Pmyo-2::YFP]}	This paper
WUM105	N2;virEx65[PHIP::RNA1WT-2]	{virEx65[PHIP::OrsayRNA1WT-1; Pmyo-2::YFP]}	This paper
WUM106	N2;virEx66[PHIP::RNA1D601A-1]	{virEx66[PHIP::OrsayRNA1D601A-1; Pmyo-2::YFP]}	This paper
WUM107	N2;virEx67[PHIP::RNA1D601A-2]	{virEx67[PHIP::OrsayRNA1D601A-1; Pmyo-2::YFP]}	This paper
WUM108	*sbp-1*(*ep79*); virEx68[PHIP::RNA1WT-1]	{virEx68[PHIP::OrsayRNA1WT-1; Pmyo-2::YFP]; *sbp-1*(*ep79*)III}	This paper
WUM109	*sbp-1*(*ep79*); virEx69[PHIP::RNA1WT-2]	{virEx69[PHIP::OrsayRNA1WT-1; Pmyo-2::YFP]; *sbp-1*(*ep79*)III}	This paper
WUM110	*sbp-1*(*ep79*); virEx70[PHIP::RNA1D601A-1]	{virEx70[PHIP::OrsayRNA1D601A-1; Pmyo-2::YFP]; *sbp-1*(*ep79*)III}	This paper
WUM111	*sbp-1*(*ep79*); virEx71[PHIP::RNA1D601A-2]	{virEx71[PHIP::OrsayRNA1D601A-1; Pmyo-2::YFP]; *sbp-1*(*ep79*)III}	This paper
WUM112	*sbp-1*(*ep79*);*sur-7*(*ku119*)	[*sbp-1*(*ep79*) III;*sur-7*(*ku119*) X]	This paper
CE541	*sbp-1*(*ep79*)	*sbp-1*(*ep79*) III	67
CE548	*sbp-1*(*ep79*) III; epEx141	epEx141 [*sbp-1*::GFP::SBP-1 + rol-6(su1006)]	68
RB2519	*drh-1*(*ok3495*)	*drh-1*(*ok3495*) IV	99
RB1716	*nhr-49*(*ok2165*)	*nhr-49*(*ok2165*) I	100
STE68	*nhr-49*(*nr2041*)	*nhr-49*(*nr2041*) I	101
RB2589	*daf-3*(*ok3610*)	*daf-3*(*ok3610*) X	102
GR1311	*daf-3*(*mgDf90*)	*daf-3*(*mgDf90*) X	103
CF1038	*daf-16*(*mu86*)	*daf-16*(*mu86*) I	104
GR1307	*daf-16*(*mgDf50*)	*daf-16*(*mgDf50*) I	105
XA7702	*mdt-15*(*tm2182*)	*mdt-15*(*tm2182*) III	106
BX165	*nhr-80*(*tm1011*)	*nhr-80*(*tm1011*) III	72
BX107	*fat-5*(*tm420*)	*fat-5*(*tm420*) V	72
BX156	*fat-6*(*tm331*);*fat-7*(*wa36*)	*fat-6*(*tm331*) IV;*fat-7*(*wa36*) V	72
VC377	*elo-5*(*gk182*)	*elo-5*(*gk182*) IV	102
VC425	*elo-6*(*gk233*)	*elo-6*(*gk233*) IV	102
MH801	*sur-7*(*ku119*)	*sur-7*(*ku119*) X	73

The transgenic RNA1 WT or dead polymerase animals were crossed by a standard method with the *sbp-1*(*ep79*) mutants and genotyped by PCR to generate the [*sbp-1*(*ep79*);PHIP::RNA1WT] and the [*sbp-1*(*ep79*);*PHIP*::*RNA1D601A*] animals. *sbp-1*(*ep79*) mutant animals with the transgenic marker were selected and challenged with Orsay virus and assayed for virus replication by qRT-PCR.

### Lipid supplementation assay.

Six-well NGM dishes were prepared with an aqueous solution of NP-40 (Sigma) to a final concentration of 0.01% and supplemented with 1 mM concentrations of any of the following reagents: stearic acid (Cayman Chemicals), oleic acid (Cayman Chemicals), linoleic acid (Cayman Chemicals), α-linoleic acid (Cayman Chemicals), γ-linoleic acid (Cayman Chemicals), stearidonic acid (Cayman Chemicals), dihomo-γ-linoleic acid (Cayman Chemicals), eicosatetraenoic acid (Cayman Chemicals), ω-3-arachidonic acid (Cayman Chemicals), arachidonic acid (Cayman Chemicals), eicosapentaenoic acid (Cayman Chemicals), C15iso (Cayman Chemicals), C17iso (Cayman Chemicals), and C13iso (Chem Cruz). Dishes without lipids were also prepared. A synchronized population of 500 L1 animals was added to the wells and incubated for 36 h. Then the animals were challenged with Orsay virus for 3 days and collected for RNA extraction and qRT-PCR. Three independent experiments in triplicate were performed.

### Zinc supplementation assay.

NAMM dishes supplemented with zinc sulfate (ZnSO_4_) or manganese chloride (Cl_2_Mn) were prepared (96). A synchronized population of 500 L1 larvae was transferred into noble agar minimal media (NAMM) dishes and infected with Orsay virus as described previously. At the end of the infection, the animals were collected for RNA extraction and quantitative PCR (qPCR) analysis.

Depletion of zinc was performed by supplementing NGM agar dishes with 1 μM *N*,*N*,*N*′,*N*′-tetrakis(2-pyridylmethyl)ethane-1,2-diamine (TPEN), a zinc-specific chelator (Sigma-Aldrich) (97).

### Epifluorescence microscopy.

The imaging of animals was carried out using a Zeiss Axio Imager M2 inverted fluorescence microscope equipped with a Hamamatsu Flash4.0 complementary metal oxide semiconductor (CMOS) camera for fluorescence. Briefly, animals were collected and anesthetized with 25 mM tetramisole and then put on a 2% agarose pad with a coverslip (5 by 5 cm) on top. Images were acquired from both fluorescence channels and bright-field channels.
