# Supplementary material for: Orsay Virus Infection of Caenorhabditis elegans Is Modulated by Zinc and Dependent on Lipids
Source: J Virol. 2022 Nov 7;96(22):e01211-22. doi: 10.1128/jvi.01211-22 (PMC9682997; doi:10.1128/jvi.01211-22)
Supplement: Supplemental file 1 — Fig. S1 to S3. Download jvi.01211-22-s0001.pdf, PDF file, 1.0 MB [file jvi.01211-22-s0001.pdf]

Supplemental material.

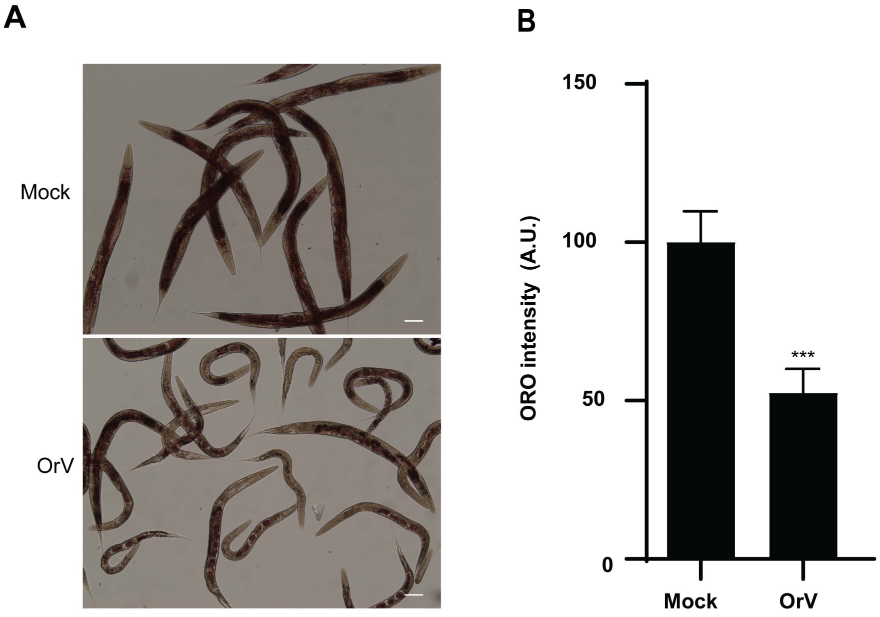

Fig. S1. Orsay virus infection reduces lipid abundance in C. elegans quantified by Oil Red O (ORO) staining. A The lipids from the *jyIs8;rde-1* strain, mock-infected or infected with Orsay virus, were stained with ORO and visualized at 48 hpi. Mock represents non-infected animals. The scale bar represents 100 µm. B) Lipid quantification was performed using imageJ as was reported before (1). The ORO intensity (shown in arbitrary units, A.U.) was normalized by setting the value of the mock infected sample as 100. Data are the arithmetic mean +/- SEM of the quantification of 30 animals. Statistically significant differences were determined by two-tailed t-test. ***P< 0.0003.

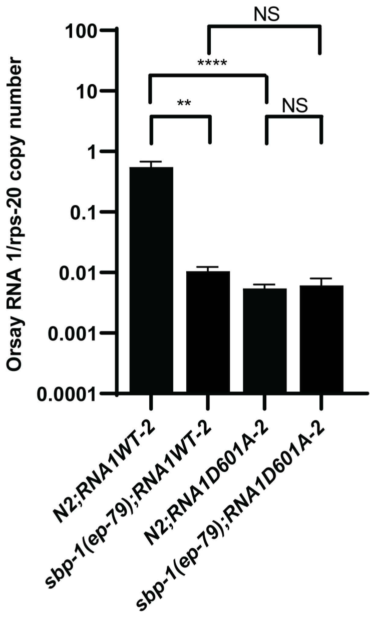

Fig. S2. An in vivo replicon system displayed that Orsay virus replication is reduced in *sbp-1*(*ep79*) mutant animals. Quantification of Orsay virus RNA1 replication from heat-induced transgenic *C. elegans* was performed by qRT-PCR. A second replicon strain was generated in the wild-type (N2) and mutant *sbp-1*(*ep79*) animal. As a negative control for replication a transgenic strain with a defective polymerase (DP) was generated in N2 and *sbp-1*(*ep79*) mutant animals. Data are the arithmetic mean +/- SEM of three independent experiments performed by triplicate. Statistically significant differences were determined by Kruskal-Wallis with a statistical difference identified between four post hoc comparisons analyzed by Dunn´s multiple-comparison test (**P < 0.0095, ****P < 0.0001). NS, not significant (P > 0.05).

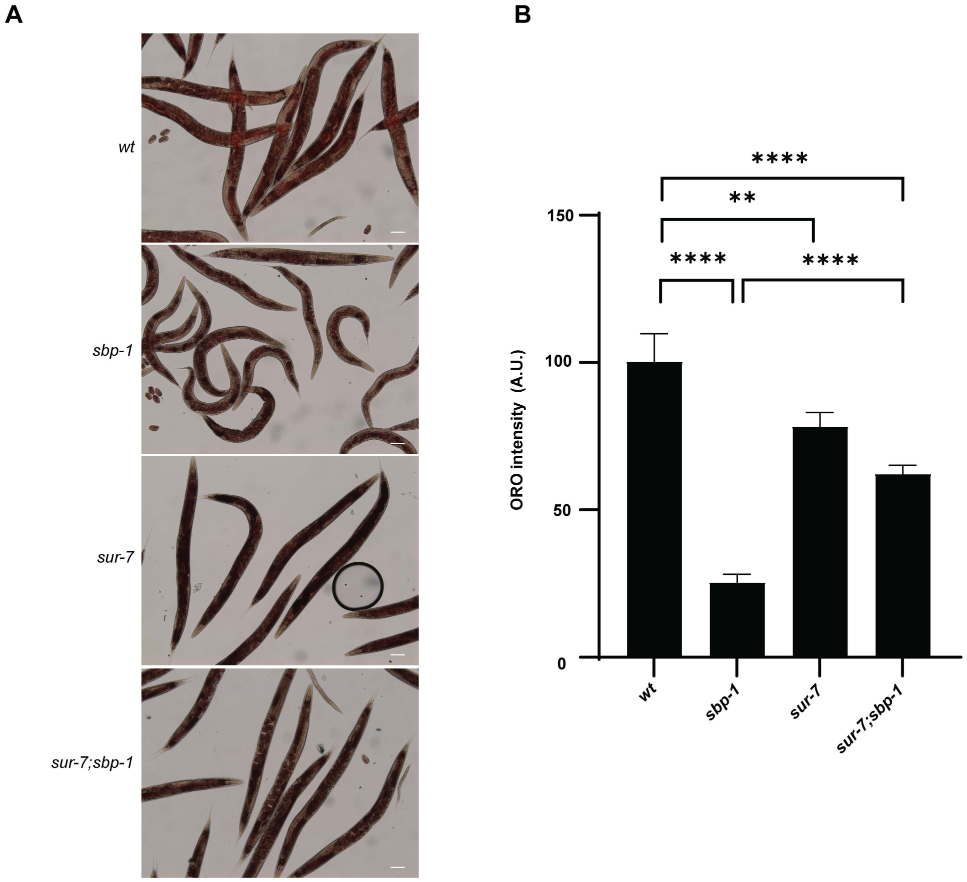

Fig. S3. Quantification of lipids in the *sur-7;sbp-1* strain by Oil Red O (ORO) staining. A The lipids from the wild type, *sbp-1*(*ep79*), *sur-7*(*ku119*) and double mutant *sur-7*(*ku119*);*sbp-1*(*ep79*) animals were stained with ORO and analyzed by microscopy. The scale bar represents 100 µm. B) Lipid quantification was performed using imageJ. The ORO intensity (shown in arbitrary units, A.U.) was normalized by setting the value of the wild type animals as 100. Data are the arithmetic mean +/- SEM of the quantification of 30 animals. Statistically significant differences were determined by one-way ANOVA with a statistical difference identified between four post hoc comparisons analyzed by Fisher´s multiple-comparison test (**P=0.007, ****P < 0.0001).

References

1. Choi LS, Shi C, Ashraf J, Sohrabi S, Murphy CT. 2021. Oleic Acid Protects Caenorhabditis Mothers From Mating-Induced Death and the Cost of Reproduction. Front Cell Dev Biol 9:690373.
